# Eicosapentaenoic acid-enriched phospholipid ameliorates insulin resistance and lipid metabolism in diet-induced-obese mice

**DOI:** 10.1186/1476-511X-12-109

**Published:** 2013-07-23

**Authors:** Xiaofang Liu, Yong Xue, Chunhua Liu, Qiaoming Lou, Jingfeng Wang, Teruyoshi Yanagita, Changhu Xue, Yuming Wang

**Affiliations:** 1 College of Food Science and Engineering, Ocean University of China, No.5 Yushan Road, Qingdao, Shandong Province, 266003, PR China; 2 Faculty of Life Science and Biotechnology, Ningbo University, No.818 Feng Hua Road, Ningbo, Zhejiang Provence, 315211, PR China; 3 Department of Health and Nutrition Science, Nishikyushu University, Saga, 842-8585, Japan

**Keywords:** Eicosapentaenoic acid, Phospholipid, Metabolic syndrome, Obesity, Insulin resistance, Lipid metabolism

## Abstract

**Background:**

Over the past two decades, a striking increase in the number of people with metabolic syndrome (MS) has taken place worldwide. With the elevated risk of not only diabetes but also cardiovascular morbidity and mortality, there is urgent need for strategies to prevent this emerging global epidemic. The present study was undertaken to investigate the effects of dietary eicosapentaenoic acid-enriched phospholipid (EPA-PL) on metabolic disorders.

**Methods:**

Male C57BL/6J mice (n = 7) were fed one of the following 4 diets for a period of 4 weeks: 1) a modified AIN-96G diet with 5% corn oil (control diet); 2) a high fat (20%, wt/wt) and high fructose (20%, wt/wt) diet (HF diet); 3) the HF diet containing 1% SOY-PL (SOY-PL diet); 4) the HF diet containing 1% EPA-PL (EPA-PL diet). The oral glucose tolerance test was performed. Plasma TG, TC, glucose, NEFA, insulin, leptin, adiponectin, TNF-α and IL-6 levels were assessed. In addition, hepatic lipid levels, lipogenic, and lipidolytic enzyme activities and gene expressions were evaluated.

**Results:**

Both EPA-PL and SOY-PL significantly inhibited body weight gain and white adipose tissue accumulation, alleviated glucose intolerance, and lowered both serum fasting glucose and NEFA levels substantially. Only EPA-PL significantly reduced serum TNF-α and IL-6 levels, and increased serum adiponectin level. EPA-PL was more effective in reducing hepatic and serum TG and TC levels than SOY-PL. Both EPA-PL and SOY-PL reduced the activities of hepatic lipogenic enzymes, such as FAS and G6PDH, but only EPA-PL significantly increased CPT, peroxisomal β-oxidation enzymes activities and CPT-1a mRNA level. Alterations of hepatic lipogenic gene expressions, such as FAS, G6PDH, ACC, SCD-1 and SREBP-1c were consistent with changes in related enzyme activities.

**Conclusions:**

According to our study, EPA-PL supplementation was efficacious in suppressing body fat accumulation, and alleviating insulin resistance and hepatic steatosis by modulating the secretion of adipocytokines and inflammatory cytokines, suppression of SREBP-1c mediated lipogenesis and enhancement of fatty acid β-oxidation. These results demonstrate that EPA-PL is a novel beneficial food component for the prevention and improvement of metabolic disorders.

## Background

Obesity and associated diseases such as type 2 diabetes dyslipidemia, and hypertension, i.e., components of the metabolic syndrome [1], are a major public health problem. While effective pharmacological interventions for the treatment of obesity-associated diseases require the use of multiple medicines and are often associated with adverse side-effects, lifestyle modifications remain essential components of treatment strategies. In this context, dietary supplementation with long-chain n-3 polyunsaturated fatty acids (n-3 PUFAs), in particular, eicosapentaenoic acid (EPA, C20:5) and docosahexaenoic acid (DHA, C22:6), present a variety of health benefits. In addition to their ability to reduce plasma lipid levels in animals [2] and humans [3], n-3 PUFAs have been shown to prevent arrhythmias, improve hypertension, and reduce platelet aggregation [4]. Dietary supplementation with n-3 PUFAs is thus beneficial for the prevention and/or treatment of cardiovascular disease [5] and possibly other inflammatory and neurological disorders [6,7]. Therefore, nutritional recommendations of 250 mg/day of EPA/DHA have been established in Western countries for n-3 PUFAs intake, to achieve nutrient adequacy and lower the n-6/n-3 PUFAs ratio [8].

Dietary EPA and DHA are provided mostly by fatty fish, where they are mainly present as esterified triglyceride (TG). In some other marine sources such as fish roe and krill, the major proportion of these n-3 PUFAs is present as esterified phospholipids (PLs). This may be biologically and therapeutically significant, because PL fatty acids are well absorbed by the intestine [9] and are readily incorporated into cell membranes [10]. Dietary PLs, particularly those originating from soy and safflower, have consistently been shown to reduce plasma and liver lipid levels in experimental animals [11]. Some recent studies have compared the potential effects of different n-3 PUFAs formulations on lipid metabolism. The supplementation of n-3 PUFAs in the form of PLs would exert superior biological and nutritional functions. These would include anti-inflammatory actions [12,13] and antioxidant activity on brain lipids [14], improved memory and learning [15], reduced blood and tissue lipids [16-19], increased bioavailability of EPA and DHA in plasma [20,21], and the tendency to reduce obesity [13,19].

The n-3 PUFA-enriched PLs used in existing studies were basically obtained from krill or fish roe, which contained 7–28% DHA and 5–12.5% EPA [12-21]. A few studies have demonstrated that DHA and EPA have different effects on lipid metabolism [22,23], vascular function [24], and inflammation [22,25] in rodents and humans. It has also been reported that EPA ethyl ester has significant hypolipidemic effects [22], but EPA in the esterified TG form has no obvious influence on lipid metabolism [23]. Thus, the effect of EPA in the PL form on metabolic disorders deserves special attention. In our preliminary study, we found that sea cucumber, *Cucumaria frondosa*, was a good natural source of EPA-enriched PL (EPA-PL), containing a large amount of EPA (more than 50% of the total fatty acids) but basically no DHA. This present study was conducted to evaluate the effects of EPA-PL on insulin resistance and hepatic steatosis in high fat/high fructose diet-induced-obese C57BL/6J mice. In addition, the molecular mechanism by which EPA-PL alters the obesity-related metabolic disorders was also investigated.

## Materials and methods

### Preparation and characterization of EPA-PL and SOY-PL

Dietary EPA-PL was extracted from the body wall of sea cucumber, *Cucumaria frondosa*. Total lipids were extracted according to the modified method of Folch *et al.*[26] and then mixed with one-fifth volume of 0.15 M NaCl solution. Then the mixture was placed into a separatory funnel and kept for 24 h to thoroughly clear the bottom (chloroform) phase. The chloroform solution was evaporated to dryness under vacuum. Then phospholipids were separated away from neutral lipids and glycolipids by a silica-gel column chromatography using chloroform, acetone and methanol sequentially as eluents. The methanol eluent was collected and EPA-PL was obtained after removal of organic solvent under vacuum. The purity of EPA-PL was confirmed to be 93.7% according to the HPLC-ELSD analysis [27]. Dietary SOY-PL (purity > 95%) was purchased from Tianjin Bodi Chemical Holding Co., Ltd. (Tianjin, China). The fatty acid compositions of the two phospholipids were determined by the method of Lou *et al.*[28] and the results are given in Table 1.

**Table 1:** Fatty acid compositions of SOY-PL and EPA-PL

**Fatty acid (%)**	**SOY-PL**	**EPA-PL**
C16:0	13.1 ± 0.36	3.85 ± 0.18
C16:1	0.36 ± 0.08	3.67 ± 0.14
C18:0	2.57 ± 0.14	9.98 ± 0.34
C18:1	8.26 ± 0.31	6.86 ± 0.16
C18:2n-6	65.6 ± 1.15	0.65 ± 0.08
C18:3n-3	10.1 ± 0.23	0.98 ± 0.06
C20:0	--	3.47 ± 0.11
C20:1	--	8.65 ± 0.34
C20:4n-6	--	6.42 ± 0.27
C20:5n-3	--	50.8 ± 1.35
C22:1	--	2.77 ± 0.09
C22:6n-3	--	1.89 ± 0.11

*Note*: “--”, none detected.

### Animals and diets

All aspects of the experiment were conducted according to guidelines provided by the ethical committee of experimental animal care at Ocean University of China (Qingdao, China). Male C57BL/6J mice aged 6 weeks were purchased from Vital River (Beijing, China). The mice were maintained in pathogen-free conditions at constant humidity of 65 ± 15% and temperature of 23 ± 2°C with a 12 h light/dark cycle. After a one-week adaptation period, the animals were divided into four groups (seven mice each): 1) CT group: a modified AIN-96G diet with 5% corn oil; 2) HF group: a high fat (20%, wt/wt) and high fructose (20%, wt/wt) diet (HF diet); 3) SOY-PL group: HF diet containing 1% SOY-PL; 4) EPA-PL group: HF diet containing 1% EPA-PL. Experimental diets were prepared according to recommendations of the American Institute of Nutrition (AIN). The ingredients and fatty acid compositions of the four experimental diets are summarized in Table 2 and Table 3. When adding SOY-PL or EPA-PL to the high fat/high fructose diet at a dose of 1%, the saturated-monounsaturated- polyunsaturated fatty acids ratios in the diets of these two phospholipid treatment groups were basically the same. After 4 weeks of feeding, mice were sacrificed after a 12 h overnight fasting. Blood was collected by orbital venipuncture and serum was separated. Liver, kidney, heart, brain and white adipose tissues were quickly excised and their weighs were obtained. All the organs and tissues were frozen in liquid nitrogen and stored at −80°C until analysis.

**Table 2:** Compositions of experimental diets

**Ingredient (g/kg)**	**CT**^***a***^	**HF**	**SOY-PL**	**EPA-PL**
Casein	245	245	245	245
Cornstarch	650	250	250	250
Fructose	--	200	200	200
Corn oil	50	50	50	50
Lard	--	200	190	190
Mineral mix	35	35	35	35
Vitamin mix	10	10	10	10
Powdered cellulose	5	5	5	5
DL-methionine	3	3	3	3
Choline bitartrate	2	2	2	2
Soybean phospholipids	--	--	10	--
EPA-enriched phospholipids	--	--	--	10

*Note*: “--”, none added; ^*a*^ modified AIN-93G.

**Table 3:** Fatty acid compositions of experimental diets

**Fatty acid (%)**	**CT**	**HF**	**SOY-PL**	**EPA-PL**
C16:0	--	26.6	25.8	25.5
C16:1	11.6	2.32	2.33	2.47
C18:0	1.31	53.6	51.1	51.4
C18:1	30.6	6.12	6.45	6.39
C18:2n-6	55.8	11.2	13.8	11.2
C18:3n-3	0.69	0.14	0.54	0.18
C20:4n-6	--	--	--	0.26
C20:5n-3	--	--	--	2.03
C22:6n-3	--	--	--	0.08
SFA	1.31	80.3	76.9	77.0
MUFA	42.2	8.44	8.78	9.32
PUFA	56.5	11.3	14.3	13.7

*Note*: *SFA* saturated fatty acid, *MUFA* monounsaturated fatty acid, *PUFA* polyunsaturated fatty acid.

### Oral glucose tolerance test

At the end of 3 weeks dietary intervention, the oral glucose tolerance test (OGTT) was performed. Following a 10 h period of feed deprivation, each mouse was intragastrically perfused with a dose of 2 g/kg body weight of 0.2 g/mL D-glucose, and then 5 μL of caudal vein blood was obtained to measure the blood glucose level using the One Touch Ultra glucometer (LifeScan, USA) at 0, 30, 60, 120 min.

### Analysis of serum parameters and hepatic lipids

The serum concentrations of TG, cholesterol (TC) and glucose were measured using enzymatic reagent kits (Biosino, China). The serum insulin (R&D system, USA), nonesterified fatty acid (NEFA, R&D system, USA), leptin (Boster Biological Technology., Ltd., China), adiponectin (Boster Biological Technology., Ltd., China), TNF-α (R&D system, USA) and IL-6 (R&D system, USA) levels were measured using enzyme-linked immunosorbent assay kits. Hepatic lipids were extracted with the modified method of Folch *et al.*[26], and the concentrations of TG and TC were analyzed with the same enzymatic kits as used in the serum analysis. Hepatic phospholipid levels were measured according to the method of Bartlett [29].

### Hepatic enzyme activity assay

A piece of liver was homogenized in six volumes of a 0.25 M sucrose solution that contained 1 mM EDTA in a 10 mM Tris–HCl buffer (pH 7.4). After the nuclei fraction was precipitated, the supernatant was centrifuged at 10 000 g for 10 min at 4°C to obtain the mitochondria fraction. The resulting supernatant was recentrifuged at 125 000 g for 60 min to precipitate microsomes, and the remaining supernatant was used as the cytosol fraction. The protein concentration was determined according to the method of Lowry *et al*. [30]. The enzyme activities of fatty acid synthase (FAS; EC2.3.1.85) [31], glucose 6-phosphate dehydrogenase (G6PDH; EC1.1.1.49) [32] and malic enzyme (ME; EC1.1.1.40) [33] in the liver cytosol fraction, mitochondrial carnitine palmitoyl transferase (CPT; EC2.3.1.21) [34] and peroxisomal β-oxidation enzyme (EC1.3.3.6) [35] were determined as described.

### Analysis of hepatic gene expression

Hepatic mRNA levels were measured by real-time polymerase chain reaction (RT-PCR). Total RNA was extracted from liver using a Trizol Reagent (Invitrogen, Japan). 1 μg total RNA were reverse transcribed into cDNA using random primer (TOYOBO, Japan). Selected genes were amplified using SYBR Green I Master Mix (Roche, Germany) in an iQ5 real-time detection system (Bio-Rad, USA) with 0.3 μM of both forward and reverse primers. PCR conditions were as follows: 1 cycle of 95°C for 10 min, 45 cycles of 95°C for 15 s, 55–60°C for 20 s and 72°C for 30 s. The purities of PCR products were assessed by melt curve analysis. Relative gene expression was quantified using the standard curve method. Results were expressed as the relative values after normalization to 18S RNA. Primer sequences were as follows: FAS (forward, 5′-TTGATGATTCAGGGAGTGG-3′; reverse, 5′-AGCAGATGAGTTGTTCTTGGAC-3′); G6PDH (forward, 5′-GTTTGGCAGCGGCAACTAA-3′; reverse, 5′-GGCATCACCCTGGTACAACTC-3′); ME (forward, 5′-TCACCTGCCCTAATGTCCCT-3′; reverse, 5′-CATGCCGTTAT CAACTTGTCC-3′); CPT-1a (forward, 5′-TCTCAGTGGGAGCGACTCT-3′; reverse, 5′-TGTGGTACACGACAATGTGCCT-3′); ACC (forward, 5′-TTGCCTATGAACTCAACAGCG-3′; reverse, 5′-AGACCATTCCGCCCATCC-3′); SCD-1 (forward, 5′-CCACTCGCCTACACCAACG-3′; reverse, 5′-GGGGTCCCTCCTCATCCT-3′); SREBP-1c (forward, 5′-AACCTCATCCGCCACCTG-3′; reverse, 5′-TGGTAGACAACAGCCGCATC-3′); PPARα (forward, 5′-TCGGAGCTGCAA GATTCAGA-3′; reverse, 5′-CAAAGCGAATTGCATTGTGTG-3′); PPARγ (forward, 5′-GTGATGGAAGACCACTCGC-3′; reverse, 5′-CCCACAGACTCGGCACTC-3′); 18S (forward, 5′-GTTGGTGGAGCGATTTGTCTG-3′; reverse, 5′-TTGCTCAATCTCGGGTGGC-3′).

### Statistical analysis

All values are expressed as means ± standard errors. Comparisons were performed with an unpaired, two-tailed Student’s *t* test or analysis of variance (ANOVA). If the overall F was significant for the latter, comparisons between means were made with Tukey’s post hoc test. *P* < 0.05 was considered statistically significant.

## Results

### General observations

There were no differences in daily food intake between dietary groups. The body weight gain of the mice in the HF group was significantly higher than that of the control group. Compared with the HF group, the EPA-PL and SOY-PL groups displayed suppressed body weight gain by 29.7% and 26.7%, respectively, and decreased white adipose tissue weights, especially for the epididymal component (*P* < 0.01 and *P* < 0.05, respectively). The mice fed the HF diet had significantly higher liver weight than the control group (*P* < 0.05), which was indicative of lipid accumulation in the liver. Meanwhile, mice fed the EPA-PL and SOY-PL diets exhibited 10.7% and 11.4% decreases in liver weights, respectively. There were no significant changes in the weights of other visceral organs (Table 4).

**Table 4:** **Growth parameters for C57BL/6J mice fed experimental diets**^***a***^

	**CT**	**HF**	**SOY-PL**	**EPA-PL**
Food intake (g/d)	2.64 ± 0.12	2.02 ± 0.12^##^	1.90 ± 0.09	1.91 ± 0.07
Body weight gain (g)	6.84 ± 0.80	8.90 ± 0.74^##^	6.52 ± 0.73**	6.26 ± 0.50**
White adipose tissue weight (g/100 g body weight)				
Epididymal	1.22 ± 0.24	2.81 ± 0.17^##^	2.25 ± 0.23*	1.90 ± 0.15**
Perirenal	1.02 ± 0.19	1.73 ± 0.21^#^	1.43 ± 0.30	1.50 ± 0.23
Mesenteric	0.43 ± 0.06	0.52 ± 0.15	0.45 ± 0.08	0.42 ± 0.08
Organ weight (g/100 g body weight)				
Liver	4.05 ± 0.33	4.48 ± 0.22^#^	3.97 ± 0.15*	4.00 ± 0.13*
Kidney	1.28 ± 0.09	1.36 ± 0.13	1.27 ± 0.09	1.28 ± 0.07
Heart	0.52 ± 0.02	0.50 ± 0.05	0.48 ± 0.03	0.47 ± 0.01
Brain	1.29 ± 0.04	1.18 ± 0.09	1.19 ± 0.06	1.24 ± 0.08

*Note*: ^*a*^ Data are presented as mean ± SEM; n = 7 mice per group. ^#^*P* < 0.05, ^##^*P* < 0.01, significant difference compared to the control group determined by Student’s *t* test. *P < 0.05, **P < 0.01, significant difference compared to the HF group determined by ANOVA (Tukey’s test).

### Oral glucose tolerance test

**Figure 1:**
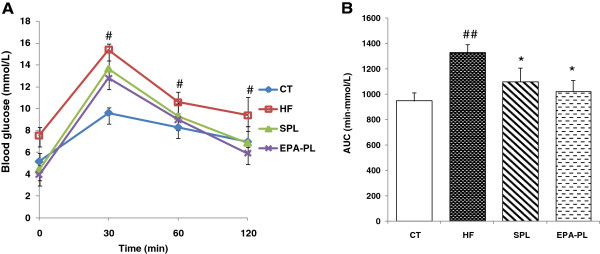
**Glucose tolerance test (A) and Area under curve (B) of C57BL/6J mice fed experimental diets**^***a***^***.****Note:*^*a*^ Data are presented as mean ± SEM; n = 7 mice per group. ^#^*P* < 0.05, ^##^*P* < 0.05, significant difference compared to the control group determined by Student’s *t* test. ^*^P < 0.05, significant difference compared to the HF group determined by ANOVA (Tukey’s test).

After 3 weeks of feeding, the mice in the HF group developed glucose intolerance as indicated by the higher area under the curve and serum glucose levels at each node of the OGTT curve (*P* < 0.05). The serum glucose levels for mice in the EPA-PL and SOY-PL groups were effectively brought down to normal levels at 60 min and 120 min, compared to those for the mice in the HF group. The decreased areas under the curve also demonstrated that EPA-PL and SOY-PL could reverse the glucose intolerance caused by the HF diet (Figure 1).

### Serum fasting glucose, insulin, NEFA, leptin, adiponectin, TNF-α and IL-6 levels

The fasting glucose and NEFA levels of the mice in the HF group were significantly increased compared to those observed in the control group (*P* < 0.01, *P* < 0.05). In comparison to the HF diet, EPA-PL and SOY-PL supplementation significantly reduced serum fasting glucose and NEFA levels. There were no significant changes in serum insulin levels (Table 5).

**Table 5:** **Serum parameters related to glucose metabolism and inflammation reaction in C57BL/6J mice fed experimental diets**^***a***^

	**CT**	**HF**	**SOY-PL**	**EPA-PL**
Glucose, mmol/L	5.18 ± 0.72	7.55 ± 0.78^##^	4.43 ± 0.34**	3.95 ± 0.29**
Insulin, ng/mL	2.24 ± 0.20	1.97 ± 0.38	1.77 ± 0.34	1.99 ± 0.11
NEFA, μmol/L	146.3 ± 9.3	173.8 ± 15.3^#^	113.8 ± 17.3**	131.3 ± 13.3*
Leptin, ng/mL	1.09 ± 0.24	2.56 ± 0.94^#^	2.37 ± 0.63	2.08 ± 0.42
Adiponectin, ng/mL	6.71 ± 0.49	8.22 ± 0.78^#^	8.90 ± 0.56	9.71 ± 0.66*
TNF-α, pg/mL	33.2 ± 3.7	52.9 ± 9.6^##^	42.1 ± 7.5	32.0 ± 6.2*
IL-6, pg/mL	20.5 ± 2.8	27.6 ± 3.1^#^	29.1 ± 5.2	19.0 ± 2.2*

*Note*: ^*a*^ Data are presented as mean ± SEM; n = 7 mice per group. ^#^*P* < 0.05, ^##^*P* < 0.01, significant difference compared to the control group determined by Student’s *t* test. *P < 0.05, **P < 0.01, significant difference compared to the HF group determined by ANOVA (Tukey’s test).

Further, we evaluated the effects of EPA-PL and SOY-PL on the serum levels of adipocytokines and inflammatory cytokines. The mice fed the HF diet displayed significantly higher serum leptin level compared to the control group (*P* < 0.05). However, neither EPA-PL nor SOY-PL had a significant effect on serum leptin levels. In comparison to the control group, serum adiponectin, TNF-α and IL-6 levels in mice from the HF group were all significantly increased (*P* < 0.05, *P* < 0.01 and *P* < 0.05, respectively). Moreover, compared to the HF diet, EPA-PL supplementation could effectively increase adiponectin level (*P* < 0.05) and reduce TNF-α and IL-6 levels (*P* < 0.05 and *P* < 0.05, respectively), while SOY-PL supplementation did not induce these parameters.

### Lipid content in serum and liver

In addition to severe insulin resistance and inflammation, HF diet-fed mice showed progressive hyperlipidemia and hepatic steatosis. Compared to the control group, the HF diet increased serum TG and TC levels by 42.7% and 36.3%, respectively. Both phospholipid treatment groups tended to have lower serum lipid levels than the HF group. In particular, EPA-PL induced significantly superior lipid lowering effects on both serum TG and TC, with a reduction of 44.1% (*P* < 0.01) and 10.3% (*P* < 0.05) compared to the HF group. In addition, EPA-PL also markedly increased HDL-c level by 23.5% (*P* < 0.05), while SOY-PL did not have this effect (Table 6).

**Table 6:** **Serum and liver Lipid contents in C57BL/6J mice fed experimental diets**^***a***^

	**CT**	**HF**	**SOY-PL**	**EPA-PL**
Serum lipids				
TG, mmol/L	0.89 ± 0.10	1.27 ± 0.13^##^	0.96 ± 0.11*	0.71 ± 0.09**
TC, mmol/L	2.34 ± 0.23	3.19 ± 0.31^##^	3.00 ± 0.23	2.86 ± 0.18*
HDL-c, mmol/L	1.21 ± 0.06	1.32 ± 0.11	1.35 ± 0.13	1.63 ± 0.08*
Hepatic lipids				
TG, mg/g	19.0 ± 2.0	35.9 ± 1.9^##^	25.7 ± 2.7**	17.5 ± 2.3**
TC, mg/g	2.66 ± 0.19	2.78 ± 0.28	2.61 ± 0.10	2.16 ± 0.17**
PL, mg/g	30.3 ± 0.8	30.1 ± 1.2	31.3 ± 0.6	32.5 ± 0.9*

*Note*: ^*a*^ Data are presented as mean ± SEM; n = 7 mice per group. ^##^*P* < 0.01, significant difference compared to the control group determined by Student’s *t* test. *P < 0.05, **P < 0.01, significant difference compared to the HF group determined by ANOVA (Tukey’s test).

Mice fed the HF diet had higher hepatic TG (*P* < 0.01) and TC levels than the control group. Mice fed both the EPA-PL and SOY-PL diets displayed significantly lowered hepatic TG levels compared to those observed in the HF group (*P* < 0.01 and *P* < 0.01, respectively). Only EPA-PL effectively decreased hepatic TC level by 22.3% (*P* < 0.01), while SOY-PL did not induce this effect (Table 6).

### Activities of hepatic enzymes related to lipid metabolism

**Figure 2:**
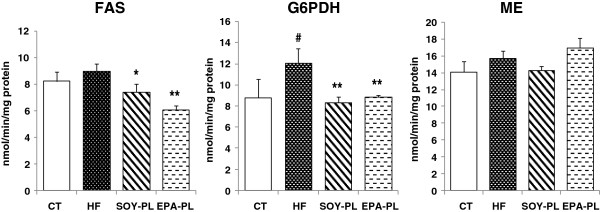
**Hepatic enzyme activities involved in fatty acid biosynthesis in C57BL/6J mice fed experimental diets**^***a***^**.***Note:*^*a*^ Data are presented as mean ± SEM; n = 7 mice per group. ^#^*P* < 0.05, significant difference compared to the control group determined by Student’s *t* test. ^*^P < 0.05, ^**^P < 0.01, significant difference compared to the HF group determined by ANOVA (Tukey’s test).

After 4 weeks feeding of the HF diet, the enzyme activities of hepatic FAS, G6PDH and ME increased in varying degrees, while EPA-PL and SOY-PL significantly reduced hepatic FAS (32.1%, *P* < 0.01; 17.1%, *P* < 0.05, respectively)and G6PDH activities (26.3%, *P* < 0.01; 31.0%, *P* < 0.01, respectively) to normal levels (Figure 2).

**Figure 3:**
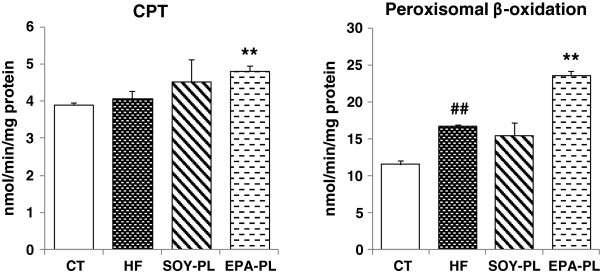
**Hepatic enzyme activities involved in fatty acid oxidation in C57BL/6J mice fed experimental diets**^***a***^**.***Note:*^*a*^ Data are presented as mean ± SEM; n = 7 mice per group. ^##^*P* < 0.01, significant difference compared to the control group determined by Student’s *t* test. ^**^P < 0.01, significant difference compared to the HF group determined by ANOVA (Tukey’s test).

In contrast, mitochondrial β-oxidation, whose rate-limiting enzyme is CPT and peroxisomal β-oxidation, were significantly enhanced in the EPA-PL group, with an improvement by 29.1% (*P* < 0.01) and 41.0% (*P* < 0.01) respectively, compared to the HF group. However, these two enzyme activities in the SOY-PL group maintained the same levels as those in the HF group (Figure 3).

### mRNA expressions of genes related to lipid metabolism in the liver

**Figure 4:**
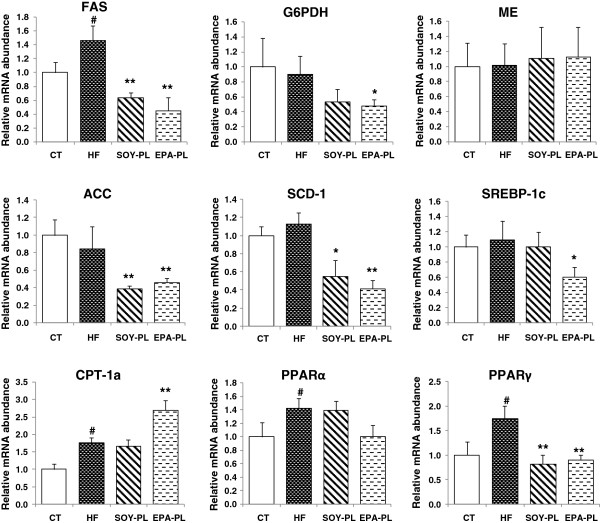
**Hepatic mRNA expressions of genes related to lipid metabolism in C57BL/6J mice fed experimental diets**^***a***^**.***Note:*^*a*^ Data are presented as mean ± SEM; n = 7 mice per group. ^#^*P* < 0.05, significant difference compared to the control group determined by Student’s *t* test. ^*^P < 0.05, ^**^P < 0.01, significant difference compared to the HF group determined by ANOVA (Tukey’s test).

Hepatic mRNA expressions of genes that regulate lipogenesis and lipolysis were examined. The mRNA expression of ME was similar among the groups, but the expressions of other lipogenesis genes, such as FAS, G6PDH, ACC, SCD-1, and SREBP-1c were markedly decreased by these two phospholipids, especially EPA-PL (Figure 4).

Nonetheless, the reduction of hepatic lipids by EPA-PL could also be a result of an upregulated mRNA expression in fatty acid β-oxidation. Therefore, we investigated the expressions of PPARα and its target gene, CPT-1a, in the liver. Although mRNA levels of PPARα were not affected by either PL, the expression of CPT-1a was greatly upregulated by EPA-PL (*P* < 0.01), while SOY-PL did not induce this effect (Figure 4).

## Discussion

The metabolic syndrome has been defined as a cluster including visceral fat obesity, impaired glucose metabolism, atherogenic dyslipidemia (high plasma triglyceride and low HDL cholesterol) and hypertension [36]. Since it raises the risk of diabetes and cardiovascular disease, there is an urgent need for strategies to prevent this emerging global epidemic. The present study showed that dietary EPA-PL supplementation effectively reduced epididymal adipose tissue weight, hyperlipidemia, and hepatic steatosis in mice fed the HF diet. In addition, EPA-PL significantly alleviated HF diet-induced glucose intolerance, increased serum adiponectin level and reduced inflammation. The biological effects of EPA-PL on lipid and glucose metabolism were superior to the common dietary SOY-PL. These data are the first to indicate that EPA-PL has beneficial effects on metabolic disorders.

Recently, it has been reported that n-3 PUFA-enriched PL extracted from marine fish tend to improve glucose intolerance in dietary obese mice [13]. In the present study, EPA-PL similarly improved HF diet-induced glucose intolerance in mice and its effect was superior to that of SOY-PL. In order to find a potential mechanism, we analyzed several serum parameters that may be related to the increase in insulin sensitivity and glucose uptake. Chronic elevation in plasma NEFA level is commonly associated with impaired insulin-mediated glucose uptake [37] and often coexists with obesity and type 2 diabetes [38]. Both EPA-PL and SOY-PL effectively inhibited the elevation of NEFA in serum, indicating that these PLs possibly ameliorate glucose metabolism in HF diet-induced-obese mice.

Adipose tissue has been recognized not only to function as a storage depot for TG but also plays an important role in regulating glucose and lipid homeostasis by secreting a variety of adipocytokines into the circulatory system. Adiponectin is the most abundant adipose-specific protein, and it plays an important role as an insulin-sensitizing adipokine whose production is decreased in obesity and in conditions associated within insulin resistance [39,40]. Several recent reports indicate that the effects of n-3 PUFA-enriched PLs on preventing excess weight gain and development of insulin resistance associated with high-fat feeding were thought to be mediated by adiponectin [13,19,41]. In agreement with these previous studies, dietary EPA-PL supplementation significantly increased serum adiponectin level in mice fed a HF diet, while SOY-PL had no such effect. We suggest that increased serum adiponectin level is the main reason for EPA-PL’s superiority to SOY-PL in the reversal of HF diet-induced glucose intolerance in mice. As for other adipocytokines, it has been known that leptin regulates energy balance by suppressing appetite [42]. In the present study, neither EPA-PL nor SOY-PL had an effect on serum leptin level and this is consistent with the unchanged food intake in each group n-3 PUFAs are known to reduce metabolic inflammation in human and rodents [43-45]. Our data show that the HF diet induced higher serum TNF-α and IL-6 levels compared to the control group. SOY-PL supplementation had no influence on the HF diet-induced inflammation. However, interestingly, pro-inflammatory secretion was not activated in the EPA-PL group. We suggest that EPA-PL is different from SOY-PL in its ability to inhibit low-grade inflammation in obese mice.

After 4 weeks of feeding, EPA-PL and SOY-PL significantly decreased liver weights and hepatic TG levels in the HF diet-fed mice. Consequently, obesity-induced fatty liver was alleviated by EPA-PL and SOY-PL. To further investigate the regulation of hepatic lipid metabolism, we analyzed the effect of dietary EPA-PL and SOY-PL on enzymes activities related to fatty acid synthesis and fatty acid β-oxidation. As shown in Figures 2 and 3, FAS and G6PDH activities were markedly suppressed by both PLs, whereas CPT and peroxisomal β-oxidation activities were only markedly enhanced by EPA-PL.

It is now well-recognized that PUFAs activate PPARα by binding directly to this transcription factor and thus stimulating hepatic fatty acid oxidation. At the same time, PUFAs inhibit hepatic fatty acid synthesis by suppressing SREBP-1 nuclear abundance through suppression of SREBP-1c gene transcription and enhancement of proteosomal SREBP-1 degradation and mRNA-SREBP-1c mRNA decay [46]. In the present study, both EPA-PL and SOY-PL supplementation resulted in a marked decrease in the gene expressions of SREBP-1c-regulated enzymes affecting fatty acid synthesis in the liver, namely, FAS, ACC, and SCD-1. This was associated with decreased levels of SREBP-1c mRNA in the EPA-PL group only. The mRNA level of the PPARα-regulated enzyme affecting fatty acid oxidation (CPT-1a) was significantly upregulated by EPA-PL. There were no obvious changes in the mRNA levels of PPARα between groups. Relevant data on expression of genes related to lipid metabolism in the liver was consistent with changes in related enzyme activities. Overall, we suggest that both EPA-PL and SOY-PL can suppress hepatic fatty acid synthesis, but only EPA-PL has the potential to effectively enhance hepatic fatty acid β-oxidation. This explains why EPA-PL exerts superior lipid metabolism improving effects in HF diet-induced obesity.

The present study also showed that EPA-PL increased serum HDL-c level and decreased hepatic cholesterol level. Previous studies have shown that fish oil suppresses the activity of 3-hydroxy-3-methyl-glutaryl-CoA reductase, a rate-limiting enzyme of cholesterol synthesis [47]. Additionally, it has been reported that dietary phosphatidylcholine decreases hepatic cholesterol levels through the enhancement of the secretion of bile cholesterol in hypercholesterolemic rabbits [48]. Further studies are necessary to clarify the effect of dietary EPA-PL on the synthesis and excretion of cholesterol in the liver.

Recent reports demonstrated that n-3 PUFA-enriched PLs obtained from krill [15] or fish roe [19] showed different biological effects from n-6 PUFA-enriched PLs (such as EGG-PL and SOY-PL). Of note, all of the n-3 PUFA-enriched PLs used in existing studies were a mixture of DHA-PL and EPA-PL [12-21]. Numerous studies have reported that DHA and EPA showed different effects on lipid metabolism [22,23], vascular function [24] and inflammation [22,25] in rodents and humans. Our present research has demonstrated that EPA-PL, which contains a large amount of EPA (more than 50% of total fatty acids) but basically no DHA, was effective in ameliorating obesity-related disorders. However, the bioactivities of DHA-PL, which contains abundant DHA but basically no EPA, have not been described. Therefore, a study comparing the biological effects of DHA-PL and EPA-PL would be of great interest.

## Conclusion

In conclusion, our results show that, compared with SOY-PL, EPA-PL exhibited superior effects on improving glucose and lipid metabolism. EPA-PL supplementation was efficacious in suppressing body fat accumulation, and alleviating insulin resistance and hepatic steatosis by modulating the secretion of adipocytokines and inflammatory cytokines, suppression of SREBP-1c mediated lipogenesis and enhancement of fatty acid β-oxidation. These findings suggest that EPA-PL could be used in the development of functional foods for the prevention of chronic metabolic diseases in humans.

## Abbreviations

ACC: Acetyl-CoA carboxylase; CPT: Carnitine palmitoyl transferase; DHA: Docosahexaenoic acid (22:6n-3); EPA: Eicosapentaenoic acid (20:5n-3); EPA-PL: EPA-enriched phospholipid; FAS: Fatty acid synthase; G6PDH: Glucose-6-phosphate dehydrogenase; HDL-c: High density lipoprotein-cholesterol; ME: Malic enzyme; MUFA: Monounsaturated fatty acid; NEFA: Nonesterified fatty acid; OGTT: Oral glucose tolerance test; PL: Phospholipids; PPAR: Peroxisome proliferator-activated receptor; PUFA: Polyunsaturated fatty acid; RT-PCR: Real-time polymerase chain reaction; SCD-1: Stearoyl-CoA desaturase-1; SFA: Saturated fatty acid; SOY-PL: Soybean phospholipid; SREBP-1c: Sterol regulatory element-binding protein-1c; TC: Total cholesterol; TG: Triglyceride

## Competing interests

The authors declare that they have no competing interest.

## Authors’ contributions

Author XFL designed and wrote a first draft of the paper. YX performed the data analysis and helped to create the tables and figures. YMW and CHX made substantial contributions to the conception and design of the study, analysis and interpretation of data and provided funding for the study. CHL, QML and XFL carried out all the experiments. JFW participated in the design of the study and statistical analysis. TY reviewed the manuscript, contributed to the final version and provided funding for the study. All authors read and approved the final manuscript.
